# Prolonged survival in patients with breast cancer and a history of brain metastases: results of a preplanned subgroup analysis from the randomized phase III BEACON trial

**DOI:** 10.1007/s10549-017-4304-7

**Published:** 2017-06-13

**Authors:** Javier Cortés, Hope S. Rugo, Ahmad Awada, Chris Twelves, Edith A. Perez, Seock–Ah Im, Patricia Gómez-Pardo, Lee S. Schwartzberg, Veronique Diéras, Denise A. Yardley, David A. Potter, Audrey Mailliez, Alvaro Moreno-Aspitia, Jin-Seok Ahn, Carol Zhao, Ute Hoch, Mary Tagliaferri, Alison L. Hannah, Joyce O’Shaughnessy

**Affiliations:** 1 Ramon y Cajal University Hospital, Madrid, Spain, and Vall d’Hebron Institute of Oncology (VHIO), Barcelona, Spain; 2 University of California, San Francisco, CA USA; 3 Medical Oncology Clinic, Jules Bordet Institute, Brussels, Belgium; 4 Leeds Institute of Cancer and Pathology and St James’s University Hospital, Leeds, UK; 5 Mayo Clinic, Jacksonville, FL USA; 6 Seoul National University Hospital, Cancer Research Institute, Seoul National University College of Medicine, Seoul, Korea; 7 Vall d’Hebron Institute of Oncology, Barcelona, Spain; 8 The West Clinic, Memphis, TN USA; 9 Institut Curie, Paris, France; 10 Sarah Cannon Research Institute, Nashville, TN USA; 11 Department of Medicine, Masonic Cancer Center, University of Minnesota, Minneapolis, MN USA; 12 Centre Oscar Lambret, Lille, France; 13 Department of Medicine, Samsung Medical Center, Sungkyunkwan University School of Medicine, Seoul, Korea; 14 Nektar Therapeutics, San Francisco, CA USA; 15 Consultant, Sebastopol, CA USA; 16 Texas Oncology-Baylor Charles A. Sammons Cancer Center/U.S. Oncology, 3410 Worth Street, Suite 400, Dallas, TX 75246 USA

**Keywords:** Brain metastases, Etirinotecan pegol, NKTR-102, Chemotherapy, Metastatic breast cancer

## Abstract

**Purpose:**

Conventional chemotherapy has limited activity in patients with breast cancer and brain metastases (BCBM). Etirinotecan pegol (EP), a novel long-acting topoisomerase-1 inhibitor, was designed using advanced polymer technology to preferentially accumulate in tumor tissue including brain metastases, providing sustained cytotoxic SN38 levels.

**Methods:**

The phase 3 BEACON trial enrolled 852 women with heavily pretreated locally recurrent or metastatic breast cancer between 2011 and 2013. BEACON compared EP with treatment of physician’s choice (TPC; eribulin, vinorelbine, gemcitabine, nab-paclitaxel, paclitaxel, ixabepilone, or docetaxel) in patients previously treated with anthracycline, taxane, and capecitabine, including those with treated, stable brain metastases. The primary endpoint, overall survival (OS), was assessed in a pre-defined subgroup of BCBM patients; an exploratory post hoc analysis adjusting for the diagnosis-specific graded prognostic assessment (GPA) index was also conducted.

**Results:**

In the trial, 67 BCBM patients were randomized (EP, *n* = 36; TPC, *n* = 31). Treatment subgroups were balanced for baseline characteristics and GPA indices. EP was associated with a significant reduction in the risk of death (HR 0.51; *P* < 0.01) versus TPC; median OS was 10.0 and 4.8 months, respectively. Improvement in OS was observed in both poorer and better GPA prognostic groups. Survival rates at 12 months were 44.4% for EP versus 19.4% for TPC. Consistent with the overall BEACON population, fewer patients on EP experienced grade ≥3 toxicity (50 vs. 70%).

**Conclusions:**

The significant improvement in survival in BCBM patients provides encouraging data for EP in this difficult-to-treat subgroup of patients. A phase three trial of EP in BCBM patients is underway (ClinicalTrials.gov NCT02915744).

**Electronic supplementary material:**

The online version of this article (doi:10.1007/s10549-017-4304-7) contains supplementary material, which is available to authorized users.

## Introduction

The rising incidence of brain metastases (BM) as a late manifestation of advanced malignancies is a major clinical problem [1–8], with a prevalence in unselected patients with metastatic breast cancer (MBC) reaching as high as 30% [2]. Depending on the breast cancer subtype, the vast majority of patients who develop BM have synchronous extra-cranial disease; consequently, effective therapeutic strategies must control intra-cranial and extra-cranial disease while maintaining or improving patients’ quality of life (QoL) [7, 8]. Indeed, in patients with breast cancer and BM (BCBM), control of systemic disease is strongly associated with improved outcomes [9–11]. Treatment options for patients with BCBM, whether de novo, recurrent following prior local surgery and/or radiotherapy, or progressive disease on radiotherapy are dismal, with small prospective trials showing modest response rates and short palliative benefit [5, 11–15].

No cytotoxic or molecularly targeted agent is approved for the treatment or prevention of BCBM [11, 12]. Molecular weight, lipophilicity, biodistribution, and drug efflux pumps all contribute to poor penetration of drugs through the blood–brain barrier and into the brain [16], although extent to which therapeutic resistance relates to inadequate drug penetration remains unclear, as does the degree to which the blood–tumor barrier is disrupted [11, 12, 15]. Current therapies have limited activity in patients with BCBM, especially those recurring post-radiation therapy [5, 11–14, 17]. This is particularly important for patients with triple-negative breast cancer (TNBC), who have a high incidence of BM and for whom there are currently no approved targeted therapies [9, 18–20].

Etirinotecan pegol (EP) is a novel long-acting topoisomerase-1 inhibitor designed to improve safety and efficacy of irinotecan by generating lower peak plasma concentrations, significantly extending the effective half-life of the SN38, the active moiety of irinotecan, from 2 to approximately 38 days [21], and concentrate deposition of the parent drug within tumor tissue. Using an experimental mouse model with established BM, a significant reduction in both the number and size of established BM and a 50% survival rate were reported for mice treated with EP; surviving animals harbored only minimal residual disease [21–23]. These findings support the ability of EP to cross the blood–tumor barrier, leading to preferential accumulation and retention in BM, followed by sustained exposure to SN38 at concentrations leading to cytotoxicity.

In the phase 3 BEACON (BrEAst Cancer Outcomes with NKTR-102) trial, patients with heavily pretreated MBC were randomized 1:1 to EP or single-agent treatment of physician’s choice (TPC) [24]. The trial allowed inclusion of patients with a history of treated, stable BM. To assess the efficacy of EP in these patients, pre-specified subgroup analyses of efficacy and safety were conducted and are reported herein. In addition, we report a post hoc analysis of survival stratified retrospectively according to the validated breast cancer-specific Graded Prognostic Assessment (GPA) index [25, 26].

## Materials and methods

### Patients

Patients eligible for the BEACON study were women (18 years or older) with an Eastern Cooperative Oncology Group (ECOG) performance status (PS) of 0 or 1; had histologically or cytologically confirmed carcinoma of the breast; measurable (by Response Evaluation Criteria in Solid Tumors [RECIST] version 1.1) [27] or non-measurable disease; prior therapy (in neoadjuvant, adjuvant and/or metastatic setting) with an anthracycline (unless not medically appropriate or contraindicated), a taxane, and capecitabine; and received between 2 and 5 prior cytotoxic regimens for locally recurrent and/or MBC, with the last dose of cytotoxic chemotherapy within 6 months of randomization. Patients with a history of BM were eligible provided their BM were symptomatically and radiologically stable, local therapy (surgery, whole brain or stereotactic radiation) had been completed, and corticosteroids for this indication had been discontinued ≥3 weeks prior to randomization. Signs and/or symptoms of BM had to have been stable for ≥28 days prior to randomization. Radiologic assessment of the brain at screening was required in patients with focal neurological signs or known BM. Patients with symptomatic or radiologic progression (according to RECIST v1.1) of BM at screening, leptomeningeal disease, or meningeal carcinomatosis were excluded.

### Study design

The study design, methodology, and results for primary and selected secondary endpoints have been previously reported [24]. A preplanned analysis was conducted in the subgroup of patients with a history of treated, stable BM at the time of study enrollment. The study was conducted according to the Declaration of Helsinki and under the principles of the International Conference on Harmonization Good Clinical Practice standards. All patients provided written informed consent, and the study was approved by relevant institutional review board or independent ethics committee.

### Administration of study treatments

EP (145 mg/m^2^) was administered every 21 days as a 90-min infusion. TPC options were defined in the protocol as single-agent eribulin, ixabepilone, vinorelbine, gemcitabine, paclitaxel, docetaxel, or *nab*-paclitaxel and administered according to local practice, with the exception of eribulin and ixabepilone, which were administered according to local product labeling. Prior to randomization, the investigator selected and centrally registered the relevant TPC agent.

### Assessments

Radiological examination was required ≤28 days prior to randomization and every 8 weeks (±7 days) thereafter until progressive disease (PD) was noted. The same imaging modality was required for subsequent radiographic assessment, whether there was measurable or non-measurable disease (RECIST v1.1). Adverse events (AEs) were assessed from the first dose of treatment until 30 days after the last dose and graded according to the National Cancer Institute (NCI) Common Toxicity Criteria for Adverse Events (CTCAE), version 4.0.

### Statistical methodology

Based on a planned sample size of 840 patients (420 patients per treatment arm), the BEACON trial had 90% power to detect a hazard ratio of 0.77 for overall survival (OS) based on death from any cause, with a two-sided alpha level of 0.049. Patients were stratified for geographical region, receptor subtype, and prior eribulin use (patients were not stratified for a history of BM). Patients with a history of treated, stable BM (BCBM subgroup) were assessed for efficacy in terms of OS (time from randomization to death from any cause), progression-free survival (PFS; time to the earliest evidence of documented disease progression as assessed by the investigator or death from any cause), and systemic objective response rate (ORR; proportion of patients with measurable disease at baseline and a confirmed complete response, partial response, stable disease, or PD by RECIST v1.1 criteria). Kaplan–Meier (KM) estimates of survival were summarized and displayed graphically; two-sided unstratified log-rank tests were used to compare OS and PFS between treatment groups. For OS (primary analysis), patients not reported as having died at the time of the data cut-off were censored at the date they were last known to be alive. Hazard ratios (HR) for EP versus TPC and corresponding 95% confidence intervals (CI) were estimated using an unstratified Cox regression model. All *P*-values reported are exploratory; no adjustments were made for the exploratory analyses in the BCBM subgroup.

Survival data were also evaluated in an exploratory post hoc analysis using the GPA index [26]. To calculate GPA, ECOG PS was converted to Karnofsky performance score (KPS) (Table S1) and receptor status (human epidermal growth factor receptor 2 [HER2], estrogen receptor, and progesterone receptor) defined tumor subtype (HER2-positive, Basal, Luminal A, Luminal B) (Table S2). GPA scores range from 0 (worst prognosis) to 4 (best prognosis) and grouped as 0–2.0 and 2.5–4.0. ORR was based on investigator-assessed measurable disease at baseline; Fischer’s exact test and Clopper–Pearson exact two-sided 95% CI were calculated for each arm accordingly. The maximum NCI CTCAE grade and frequency of AEs were compared between the BCBM treatment groups. AEs were summarized for patients who received at least one study drug dose. Odds ratios comparing EP versus TPC were calculated for selected AEs occurring in ≥10% of patients.

## Results

Of the 852 patients randomized in the BEACON trial at 135 medical centers between December 2011 and August 2013, 67 patients had a history of treated, stable BM (EP arm, *n* = 36; TPC arm, *n* = 31). Of these, 19 patients randomized to EP and 18 randomized to TPC had radiologically detectable BM at study entry. Of the 67 patients, 61 (91%; 34 out of 36 patients in EP and 27 out of 31 patients in TPC) had received prior radiotherapy to their BM; 11 patients (16%) had undergone surgical resection, most in combination with radiotherapy. Time from initial BM diagnosis was similar between the two groups: 0.91 and 0.58 years for the EP and TPC groups, respectively. Median time since last brain-directed radiotherapy to first study treatment was also similar between the two groups: 7.8 and 6.7 months for the EP and TPC groups, respectively.

As denoted in Table 1, BCBM patients had similar patient and disease characteristics at baseline to those of the overall BEACON intention-to-treat (ITT) population. In BCBM patients, critical baseline prognostic features were balanced between the two groups (including central nervous system (CNS)-directed therapy, patients with TNBC, median time since diagnosis of breast cancer and a diagnosis of BM, and GPA score). Some marginal differences in baseline features observed baseline ECOG PS 0 (30.6% of patients in EP arm vs. 16.1% in TPC arm) and liver involvement (72.2% vs. 58.1% in the EP and TPC arms, respectively). The median number of days of study drug exposure for patients in the BCBM population was similar between treatment arms (47.5 days for EP and 44 days for TPC); both treatment arms received a median number of three cycles. The maximum number of study drug cycles was 23 for EP and 13 for TPC. Of the patients receiving TPC, the majority received multiple (weekly) infusions in each treatment cycle (four patients received a single infusion every 3 weeks).

**Table 1:** Demographics and baseline patient characteristics

	BMH		ITT
	Etirinotecan pegol (*n* = 36)	TPC (*n* = 31)	Total (*n* = 852)
Demographics			
Age (years), median	54.5	54.0	55.0
Range	28–75	37–76	28–84
ECOG PS, baseline			
0	11 (30.6%)	5 (16.1%)	309 (36.3%)
1	25 (69.4%)	25 (80.6%)	537 (63.0%)
2	0	1 (3.2%)	5 (0.6%)
3	0	0	1 (0.1%)
Cancer history			
Time since BC diagnosis (years), median	4.4	5.2	5.6
Time since LR/MBC diagnosis (years), median	2.6	2.4	2.5
Initial disease-free interval (years), median	2.3	3.1	2.7
Time since brain metastases diagnosis (years)	0.91	0.58	NA
Visceral disease at enrollment	30 (83.3%)	27 (87.1%)	643 (75.5%)
Metastatic involvement at study entry			
Bones	27 (75.0%)	13 (41.9%)	489 (57.4%)
Brain	19 (52.8%)	18 (58.1%)	37 (4.3%)
Liver	26 (72.2%)	18 (58.1%)	456 (53.5%)
Lung	15 (41.7%)	15 (48.4%)	323 (37.9%)
Hormone receptor status			
Positive (ER+ or PR+)	25 (69.4%)	21 (67.7%)	585 (68.7%)
Negative	11 (30.6%)	10 (32.3%)	266 (31.2%)
HER2/neu receptor status			
Positive	4 (11.1%)	5 (16.1%)	62 (7.3%)
Negative	32 (88.9%)	26 (83.9%)	782 (91.8%)
Triple negative	10 (27.8%)	8 (25.8%)	236 (27.7%)
Prior therapy			
Number of prior regimens for MBC, median	3.0	3.0	3.0
Anthracycline Refractory^a^	34 (94.4%) 6 (16.7%)	30 (96.8%) 3 (9.7%)	816 (95.8%) 115 (13.5%)
Taxane Refractory^a^	36 (100.0%) 18 (50.0%)	31 (100.0%) 13 (41.9%)	852 (100.0%) 343 (40.3%)
Capecitabine Refractory^a^	36 (100.0%) 26 (72.2%)	31 (100.0%) 19 (61.3%)	852 (100.0%) 624 (73.2%)
Eribulin	7 (19.4%)	9 (29.0%)	143 (16.8%)
Hormonal therapy	25 (69.4%)	19 (61.3%)	609 (71.5%)
HER2-directed therapies^b^	6 (16.7%)	5 (16.1%)	87 (10.2%)
Prior RT to brain	34 (94.4%)	27 (87.0%)	

	Etirinotecan pegol		TPC	
	BMH (*n* = 34)	ITT (*n* = 425)	BMH (*n* = 27)	ITT (*n* = 406)
Drug exposure				
Therapy received in TPC				
Eribulin			8 (29.6%)	164 (40.4%)
Vinorelbine			5 (18.5%)	94 (23.2%)
Gemcitabine			9 (33.3%)	71 (17.5%)
*nab*-Paclitaxel			3 (11.1%)	31 (7.6%)
Paclitaxel			0	18 (4.4%)
Ixabepilone			1 (3.7%)	15 (3.7%)
Docetaxel			1 (3.7%)	13 (3.2%)
Exposure duration (days), median (range)	47.5 (1–540)	48 (1–766)	44 (1–376)	56.5 (1–607)
Number of cycles completed, median (range)	3 (1–23)	3 (1–35)	3 (1–13)	3 (1–26)

	BMH		
	Etirinotecan pegol (*n* = 36)	TPC (*n* = 31)	Total (*n* = 67)
Graded prognostic assessment (GPA) index			
Score			
0–2	13 (36.1%)	10 (32.3%)	23 (34.3%)
2.5–4	23 (63.9%)	21 (67.7%)	44 (65.7%)
Median/mean score	2.3/2.5	2.3/2.5	2.3/2.5

*BC* breast cancer, *BMH* history of treated, stable breast cancer brain metastases, *ECOG* Eastern Cooperative Oncology Group, *ER* estrogen receptor, *GPA* graded prognostic assessment, *HER2* human epidermal receptor type 2, *ITT* intention-to-treat, *LR* locally recurrent, *MBC* metastatic breast cancer, *PR* progestin receptor, *PS* performance status, *RT* radiation therapy, *TPC* treatment of physician’s choice

^a^Defined as disease progression while receiving therapy in the metastatic setting within 8 weeks of the last dose of the last regimen

^b^Included trastuzumab, lapatinib, pertuzumab, and TDM1

### Efficacy

**Fig. 1:**
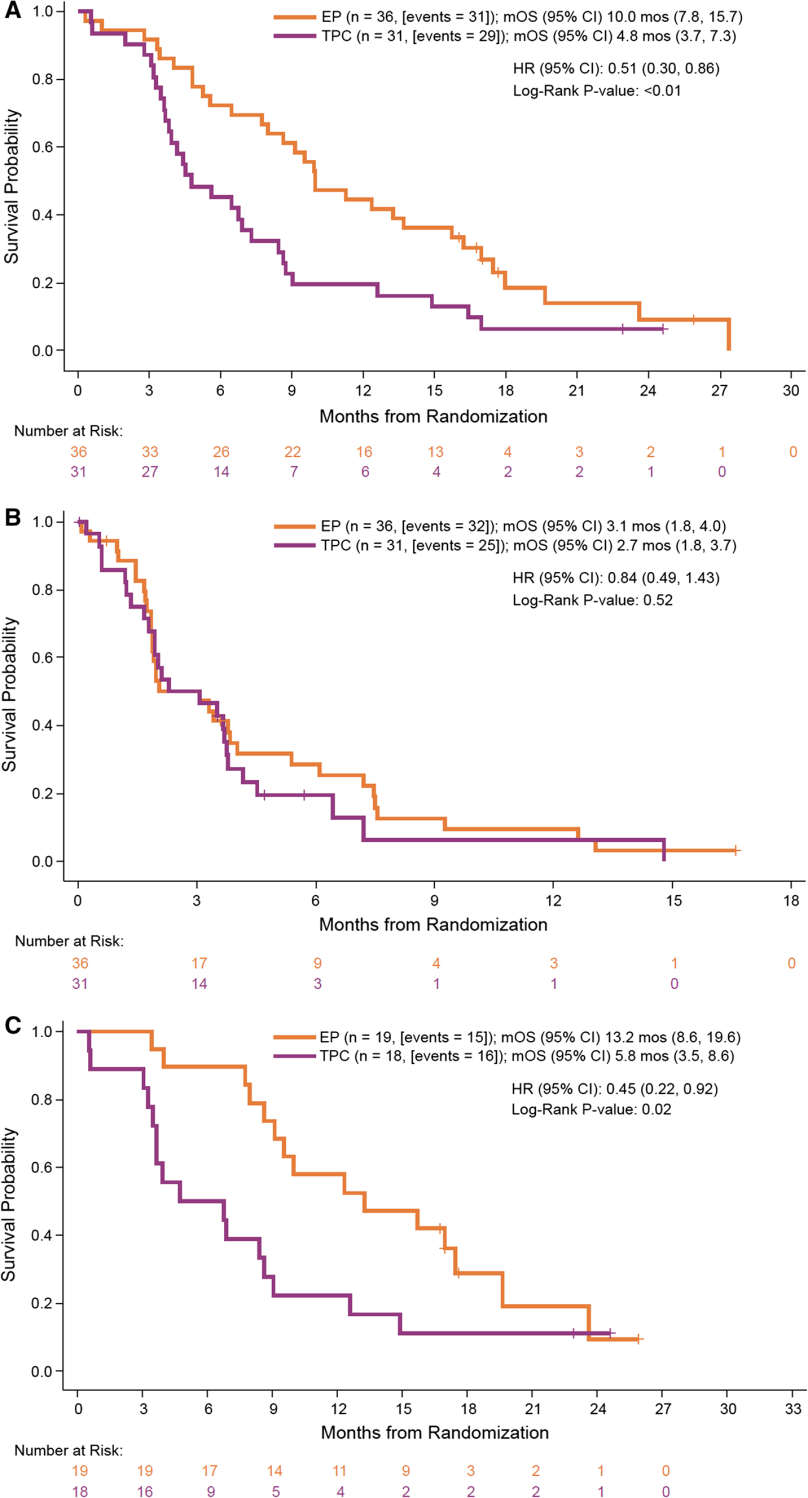
Kaplan–Meier estimates for **a** overall survival and **b** progression-free survival for patients with stable, treated brain metastases; and **c** overall survival for patients with radiologically detectable, but stable, brain lesions at study entry. *CI* confidence interval, *HR* hazard ratio, *mOS* median overall survival, *mPFS* median progression-free survival, *TPC* treatment of physician’s choice

With a median follow-up of 21.1 months for the EP arm and 21.7 months for the TPC arm in the primary survival analysis, a total of 60 deaths occurred in the 67 BCBM patients; 31 (86.1%) in the EP arm and 29 (93.5%) in the TPC arm. Median OS was 10.0 months (95% CI 7.8–15.7 months) versus 4.8 months (95% CI 3.7–7.3 months) for patients randomized to EP and TPC, respectively (Table 2); KM-curves are shown in Fig. 1, demonstrating a HR of 0.51 (95% CI 0.30–0.86) favoring EP. Overall survival results favored EP regardless of type of prior BM therapy [for surgery, patients randomized to EP had a median OS of 13.5 months compared to 3.2 months for TPC (HR 0.38); for radiotherapy, 10.0 and 5.1 months, respectively (HR 0.56)] or tumor subtype [HER2-positive, 16.1 vs. 8.6 months (HR 0.55); TNBC, 6.7 vs. 3.8 months (HR 0.27); and hormone receptor-positive, 12.2 vs. 5.2 months (HR 0.47)].

**Table 2:** Efficacy

	Etirinotecan pegol (*n* = 36)	TPC (*n* = 31)	*P*-value
BMH subgroup			
Objective response rate (systemic)	5 (15.6%)	1 (3.7%)	0.20
Evaluable population^a^	*n* = 32	*n* = 27	
95% CI	5.3–32.8	0.1–19.0	
Complete response	0	0	
Partial response	5 (15.6%)	1 (3.7%)	
Stable disease	9 (28.1%)	9 (33.3%)	
Progressive disease	14 (43.8%)	9 (33.3%)	
Not evaluable	4 (12.5%)	8 (29.6%)	
Overall survival (months)			
Median	10.0	4.8	<0.01
95% CI	7.8–15.7	3.7–7.3	
6-month OS rate	72.2%	45.2%	
12-month OS rate	44.4%	19.4%	
Progression-free survival (months)			
Median	3.1	2.7	0.52
95% CI	1.8–4.0	1.8–3.7	
3-month PFS rate	50.1%	50.0%	
6-month PFS rate	28.6%	19.5%	

	Etirinotecan pegol (*n* = 19)	TPC (*n* = 18)	*P*-value
Radiologically detectable brain lesions at study entry			
Objective response rate (systemic)	4 (25%)	1 (6.3%)	0.33
Evaluable population^a^	*n* = 16	*n* = 16	
95% CI	7.3–52.4	0.2–30.2	
Complete response	0	0	
Partial response	4 (25.0%)	1 (6.3%)	
Stable disease	5 (31.3%)	6 (37.5%)	
Progressive disease	6 (37.5%)	4 (25.0%)	
Not evaluable	1 (6.3%)	5 (31.3%)	
Progressive disease in brain lesion	6 (37.5%)	6 (37.5%)	
Overall survival (months)			
Median	13.2	5.8	0.02
95% CI	8.6–19.6	3.5–8.6	
6-month survival rate	89.5%	50.0%	
12-month survival rate	57.9%	22.2%	

OS by GPA category—BMH Subgroup	Etirinotecan pegol (*n* = 36)	TPC (*n* = 31)	*P*-value
0–2			
n	13	10	
Median, months	7.8	3.8	<0.01
2.5–4			
n	23	21	
Median, months	13.2	6.9	0.06

OS by GPA category—radiologically detectable brain lesions at baseline	Etirinotecan pegol (*n* = 19)	TPC (*n* = 18)	*P*-value
0–2			
n	6	5	
Median, months	9.6	3.5	
2.5–4			
n	13	13	
Median, months	16.8	6.9	

^a^Efficacy evaluable population (measureable systemic disease at baseline required)

*BMH* history of treated, stable breast cancer brain metastases, *CI* confidence interval, *GPA* graded prognostic assessment, *OS* overall survival, *PFS* progression-free survival, *SD* stable disease, *TPC* treatment of physician’s choice

**Fig. 2:**
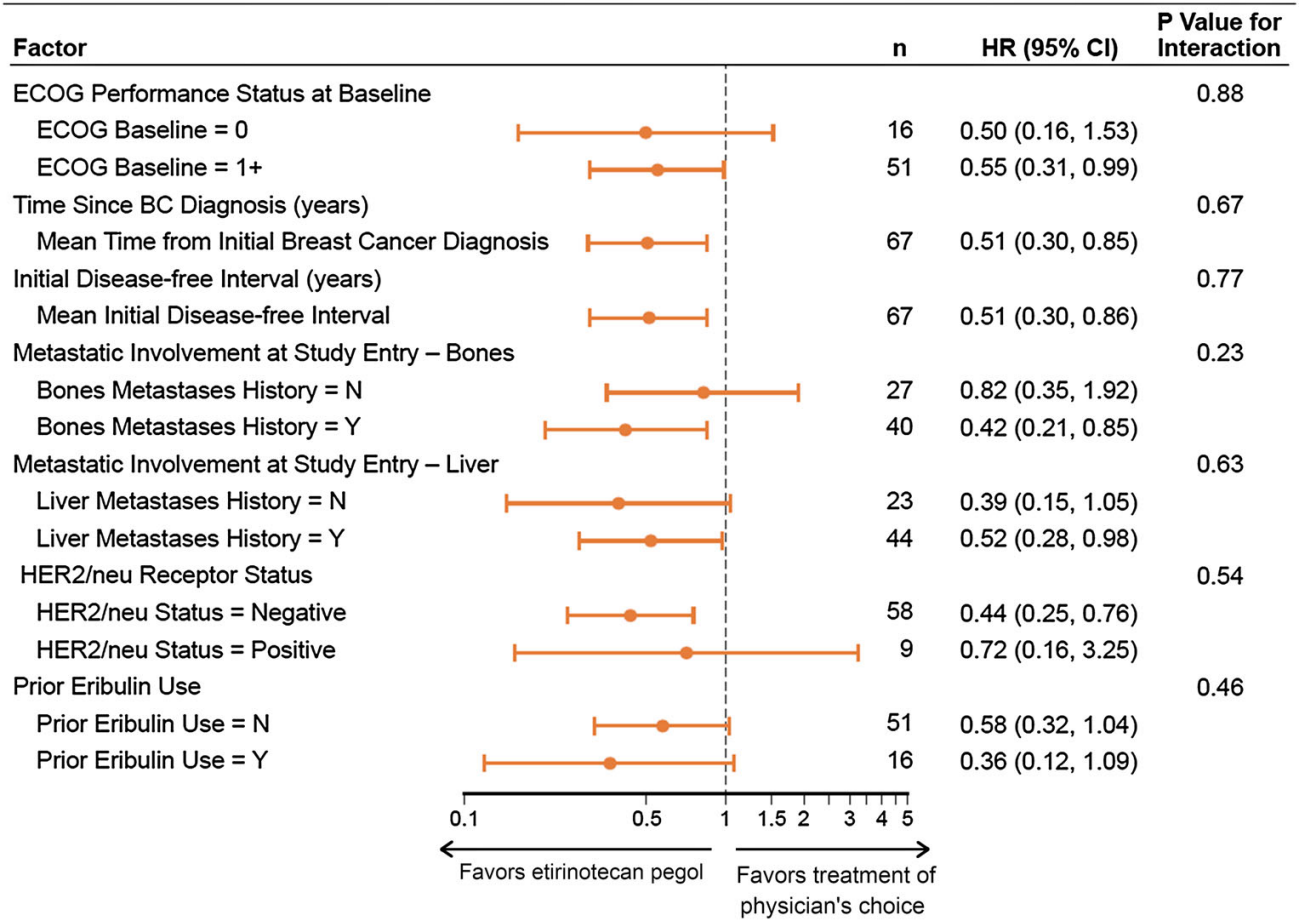
Forest plot of hazard ratios (HR) with 95% confidence intervals (CI) for overall survival for selected prognostic factors in the intention-to-treat (ITT) population with a history of treated, stable brain metastases. *BC* breast cancer, *CI* confidence interval, *ECOG* Eastern Cooperative Oncology Group, *HER2* human epidermal growth factor receptor 2, *HR* hazard ratio, *TPC* treatment of physician’s choice

Considerable improvements in 6- and 12-month survival rates were also associated with EP treatment. The 6-month rates were 72.2 and 45.2% for EP and TPC, respectively; corresponding 12-month rates were 44.4 and 19.4%, respectively. Forest plot of HRs, with corresponding 95% CIs and *P* value, for OS in preselected prognostic factors is shown in Fig. 2. As depicted, there was consistency of benefit across all subgroups favoring EP treatment. In those patients with radiologically detectable but stable and treated BM on baseline imaging, median OS was 13.2 months for EP (*n* = 19) versus 5.8 months for TPC (*n* = 18) (HR 0.45; 95% CI 0.22–0.92) (Fig. 1). The proportion of patients alive at 6 and 12 months were 89.5 versus 50%, and 57.9 versus 22.2% for the EP and TPC arms, respectively. The median PFS was 3.1 months for EP and 2.7 months for TPC (HR 0.84; 95% CI 0.49–1.43; *P* = 0.52) (Fig. 1). PFS rates at 3 months were similar between arms at 50%; 6-month PFS was 28.6 and 19.5% in the EP and TPC arms, respectively (Table 2).

All BCBM patients had at least one extra-cranial site of metastasis at baseline: 66% had liver metastases and 72% had 3 or more sites of metastatic disease. Among BCBM patients who had measurable systemic disease at baseline (EP, *n* = 32; TPC, *n* = 27), 5 (15.6%) had a systemic ORR in the EP arm compared with 1 (3.7%) patient in TPC group (Table 2); all were partial responses. No intra-cranial responses were seen. Of the remaining patients, approximately one-third in each arm had stable disease. For the five patients with a response in the EP arm, median response duration was 5.6 months; response duration in the single responder in the TPC group was 3.7 months.

### Graded prognostic assessment index

The treatment groups were well balanced for GPA indices (KPS, tumor subtype, and age) at baseline. Of the 67 patients, 23 had low (0–2; i.e., poorer prognosis) GPA scores and 44 had higher (2.5–4; i.e., better prognosis) scores. Thirty-six percent of patients in the EP arm had low GPA scores versus 32% for the TPC arm; both mean and median GPA scores for the treatment groups were the same (mean 2.3, median 2.5; Table 1). The median OS for patients with a GPA of 0–2 was 7.8 months in the EP arm and 3.8 months in the TPC arm (HR 0.27;95% CI 0.10–0.72; *P* < 0.01) (Table 2). The median OS for patients with a GPA of 2.5–4 was 13.2 months for EP and 6.9 months for TPC (HR 0.54; 95% CI 0.28–1.04; *P* = 0.062). The HR for OS of the 67 patients after adjusting for the two GPA groups was 0.44, favoring EP.

The same analyses were conducted for patients who had radiologically detectable but stable BM on baseline imaging. In this smaller group (EP, *n* = 19; TPC, *n* = 18), the same trend was seen. The median OS for patients with a GPA of 0–2 was 9.6 months for EP and 3.5 months for TPC; median OS for patients with a GPA of 2.5–4.0 was 16.8 and 6.9 months, respectively.

### Safety

Sixty-one patients comprised the safety population, 34 in the EP arm and 27 in the TPC arm (2 and 4 patients, respectively, were randomized but did not proceed to treatment due to withdrawal of consent or rapid deterioration of PS). The proportion of patients who experienced at least one grade 3 or higher treatment-emergent AE was lower in the EP arm compared with TPC (50% vs. 70.4%, respectively; Table 3). Neutropenia, the most common grade ≥3 AE, occurred in 33.3% of TPC patients versus 14.7% EP patients. The incidence of grade 3 diarrhea was nearly identical in the two study arms, 5.9 versus 3.7% in the EP and TPC arms, respectively. Treatment discontinuation was attributed to an AE in seven patients in the EP arm (neutropenia or neutrophil count, *n* = 3; diarrhea, *n* = 2; ascites, *n* = 1; and vomiting, *n* = 1) and 1 patient in the TPC arm (confusional state, *n* = 1).

**Table 3:** Common grade 3 or higher adverse events

	Etirinotecan pegol		TPC	
	BMH (*n* = 34)	ITT (*n* = 425)	BMH (*n* = 27)	ITT (*n* = 406)
Number of patients with at least one AE grade 3 or higher	17 (50.0%)	204 (48.0%)	19 (70.4%)	256 (63.1%)
Hematologic				
Neutropenia-related events	5 (14.7%)	41 (9.6%)	9 (33.3%)	125 (30.8%)^b^
Anemia	1 (2.9%)^a^	20 (4.7%)^a^	1 (3.7%)	19 (4.7%)
Non-hematologic				
Diarrhea	2 (5.9%)^a^	41 (9.6%)^a^	1 (3.7%)^a^	5 (1.2%)^a^
Nausea	2 (5.9%)^a^	15 (3.5%)^a^	0	8 (2.0%)^a^
Pleural effusion	2 (5.9%)	15 (3.5%)	0	16 (3.9%)
Dehydration	1 (2.9%)^a^	17 (3.5%)^a^	1 (3.7%)^b^	8 (2.0%)^b^
Hypokalemia	1 (2.9%)	10 (2.4%)	1 (3.7%)	7 (2.0%)
Hyponatremia	0	3 (<1%)^a^	2 (7.4%)	8 (2.0%)
Neuropathy-related events	0	2 (<1%)	0	15 (3.7%)^a^

*AE* adverse event, *BMH* history of treated, stable breast cancer brain metastases, *ITT* intention-to-treat, *TPC* treatment of physician’s choice

^a^No grade 4 reported

^b^Grade 5 event(s) reported

In the BEACON ITT population, a longitudinal analysis using repeated measure linear mixed model in change from baseline over 32 weeks showed that EP was statistically superior (*P* < 0.02) in the treatment difference for global health status and physical functioning and numerically superior in all other functions. The mean difference between treatment groups was larger in the BCBM patients, although the sample size was too small to detect statistical significance (*P* > 0.05).

### Post-study treatment

In the BCBM subgroup, 72.2% of those randomized to EP received at least one subsequent cancer therapy versus 48.4% randomized to TPC. Eribulin and gemcitabine were the most commonly prescribed follow-on therapies in patients randomized to EP (41.7 and 27.8%, respectively); the most commonly prescribed subsequent therapies in the TPC arm were paclitaxel (12.9%) and cyclophosphamide (12.9%). Use of eribulin (combining those patients who had received eribulin prior to study, as part of the TPC group or as follow-on therapy) was similar between the two groups. For the EP group, 7 (19.4%) patients received eribulin prior to study and 15 (41.7%) patients as a follow-on therapy. For the TPC group, 9 (29.0%) patients received prior eribulin; 8 (25.8%) patients had eribulin as their TPC agent, and 2 (6.5%) as follow-on therapy.

## Discussion

In the overall BEACON study, EP was associated with a 2.1-month improvement in OS compared to TPC; however, statistical significance was not reached (HR 0.87; 95% CI 0.75, 1.02; *P* = 0.08) [24]. In a preplanned subgroup analysis of patients with a history of stable, treated BM, EP demonstrated a substantial reduction in the risk of death (HR 0.51) compared to conventional chemotherapy. Median survival was improved by 5.2 months (10.0 vs. 4.8 months), with a doubling of 12-month survival rate (44 vs. 20%). Findings were even more pronounced in the small subset of patients with radiologically detectable, but stable, brain lesions at baseline, with a 7.4-month survival advantage for those patients receiving EP.

In a post hoc evaluation using the GPA Index as described herein, treatment with EP was associated with improved OS for patients in both better and worse prognosis groups, reinforcing the activity of EP in BCBM patients. The GPA tool, which assigns scores for significant prognostic indices of KPS, tumor subtype, and age, was originally developed to predict prognosis in patients with newly diagnosed BM [25, 26]. It should be noted that many of the BCBM patients in this analysis were not newly diagnosed with BM; however, the GPA analysis provided a way to stratify patients to correct for potential imbalances between the groups, most notably the differences in HER2 and performance status.

All BCBM patents randomized into BEACON had extra-cranial disease, the majority of whom (72%) had a high burden of systemic disease (defined as three or more sites of metastases). This is consistent with the rarity of CNS lesions being the solitary site of disease in MBC, occurring in fewer than 5% [28]. Most BCBM patients die from progression of systemic (extra-cranial) disease or a combination of extra-cranial and intra-cranial progression. In one series of 83 patients with BCBM, only 15% died of isolated CNS disease progression with stable systemic disease at the time of death [29]. Hence, control of both intra-cranial and extra-cranial disease is crucial. No intra-cranial objective responses were seen in BCBM patients randomized to either EP or TPC; however, two patients in the EP arm had non-target CNS lesions (present at baseline) become absent during their course of treatment. Of note, BCBM patients in the BEACON study were required to have had all CNS lesions treated (with either radiotherapy or surgery) and no evidence of radiographic progression or neurological symptoms prior to randomization. As such, all brain lesions present at study entry were considered non-target lesions by RECIST and best overall “in-brain response” could therefore be a complete response, “non-CR, non-PD,” or progression. The ongoing phase three trial in BCBM patients (ClinicalTrials.gov NCT02915744) uses the more recently introduced RANO-BR criteria [30], which assesses intra-cranial and extra-cranial disease independently for both response and progression.

Unselected, retrospective, historical data indicate that median survival of patients with brain metastases from breast cancer after radiation therapy is approximately 4–6 months [31–33], and varies depending on prognostic factors from 3.4 months to 2 years [25, 26]. We acknowledge that the results reported herein are in a highly selected patient population and that the lack of systematic brain assessment is a limitation of the study (head imaging was only required at baseline and follow-up for patients with focal neurological deficits or a known history of brain metastases). However, as a recent review of the literature emphasizes, there is a relative paucity of data in this patient population, with only small prospective trials evaluating chemotherapy in patients with BCBM previously treated with either systemic therapy or radiotherapy [11]. The biological rationale for EP accumulation in brain metastases is strong, with results of this study providing solid hypothesis generation. The activity of single-agent EP against intra-cranial malignancies is supported by a phase 2 trial in which 3 of 18 patients with glioblastoma multiforme (GBM) progressing after bevacizumab treatment had confirmed partial responses according to RANO criteria, corresponding to a 17% response rate; two of the responses were highly durable, lasting ≥19 months [34]. To place this in context, it is rare to see objective responses in patients with GBM whose disease has progressed on bevacizumab as evidenced by phase II trials [35–47]. The plausibility of an enhanced survival effect using EP is further strengthened by the non-clinical pharmacology data in mouse models of human tumors including the CNS, comparing EP to either conventional irinotecan or to the TPC agents used in BEACON. The data from two separate studies support differential distribution and markedly longer retention of EP and SN38 active metabolite, with resultant longer survival in mice treated with EP, 100-fold higher brain concentrations and resolution of brain lesions upon necropsy [22, 23].

As a topoisomerase-I inhibitor, SN38 derived from EP has a mechanism of action and a toxicity spectrum that is distinct from that of the tubulin-inhibitor cytotoxic drugs, which comprises most of the standard of care chemotherapies for MBC treatment. In patients with advanced malignancies who have received multiple prior regimens, an alternative mechanism of action is important: it reduces the likelihood of cross-resistance and contribution to cumulative toxicities. In the BEACON trial [24, 48] and the BCBM subgroup, EP demonstrated a lower rate of grade 3 and dose-limiting/QoL-reducing toxicities associated with tubulin-inhibitors (notably neuropathy, myelosuppression, fatigue, cardiomyopathy, and alopecia), although EP was associated with more gastrointestinal toxicities, including diarrhea.

There remains a critical need for therapeutic interventions that prolong patient survival and maintain or improve QoL of patients with breast cancer and brain metastases. Despite the relatively small number of patients in this preplanned subgroup analysis, the clear survival benefit and favorable safety profile demonstrated over that of commonly prescribed agents in this setting, together with phase II evidence of single-agent activity in recurrent high-grade primary brain tumors, support further study of EP as treatment of brain metastases for SN38-sensitive primary tumors including breast cancer. An international phase three trial in this population is underway (ClinicalTrials.gov NCT02915744). Nektar Therapeutics submitted a marketing authorization application for conditional approval of EP in Europe for the treatment of adult patients with breast cancer and brain metastases. The decision regarding conditional approval is expected in 2017.

## Electronic supplementary material

Below is the link to the electronic supplementary material.

Supplementary material 1 (DOCX 16 kb)

## Abbreviations

AE: Adverse events
BC: Breast cancer
BCBM: Breast cancer and brain metastases
BEACON: BrEAst cancer outcomes with NKTR-102
BM: Brain metastases
BMH: History of treated, stable breast cancer brain metastases
CI: Confidence interval
CNS: Central nervous system
CTCAE: Common toxicity criteria for adverse events, version 4.0
ECOG: Eastern Cooperative Oncology Group
EP: Etirinotecan pegol
ER: Estrogen receptor
GBM: Glioblastoma multiforme
GPA: Graded prognostic assessment
HER2: Human epidermal growth factor receptor 2
HR: Hazard ratio
ITT: Intention-to-treat
KM: Kaplan–Meier
KPS: Karnofsky performance score
LR: Locally recurrent
MBC: Metastatic breast cancer
MMRM: Repeated measure linear mixed model
NCI: National Cancer Institute
ORR: Objective response rate
OS: Overall survival
PD: Progressive disease
PFS: Progression-free survival
PR: Progesterone receptor
PS: Performance status
RECIST: Response evaluation criteria in solid tumors
RT: Radiation therapy
TPC: Treatment of physician’s choice
