# Supplementary material for: Prolonged survival in patients with breast cancer and a history of brain metastases: results of a preplanned subgroup analysis from the randomized phase III BEACON trial
Source: Breast Cancer Res Treat. 2017 Jun 13;165(2):329–41. doi: 10.1007/s10549-017-4304-7 (PMC5543189; doi:10.1007/s10549-017-4304-7)
Supplement: Supplementary file 1 — Supplementary material 1 (DOCX 16 kb) [file 10549_2017_4304_MOESM1_ESM.docx]

**Supplementary Material**

**Prolonged survival in patients with breast cancer and a history of brain metastases: Results of a preplanned subgroup analysis from the randomized phase III BEACON trial**

**Table S1** Performance status conversion

| **ECOG Performance Status** | **Karnofsky Performance Status** |
| --- | --- |
| 0—Fully active, able to carry on all pre-disease performance without restriction | 100—Normal, no complaints; no evidence of disease  90—Able to carry on normal activity; minor signs or symptoms of disease |
| 1—Restricted in physically strenuous activity but ambulatory and able to carry out work of a light or sedentary nature, e.g., light house work, office work | 80—Normal activity with effort, some signs or symptoms of disease  70—Cares for self but unable to carry on normal activity or to do active work |
| 2—Ambulatory and capable of all selfcare but unable to carry out any work activities; up and about more than 50% of waking hours | 60—Requires occasional assistance but is able to care for most of personal needs  50—Requires considerable assistance and frequent medical care |
| 3—Capable of only limited selfcare; confined to bed or chair more than 50% of waking hours | 40—Disabled; requires special care and assistance  30—Severely disabled; hospitalization is indicated although death not imminent |
| 4—Completely disabled; cannot carry on any selfcare; totally confined to bed or chair | 20—Very ill; hospitalization and active supportive care necessary  10—Moribund |
| 5—Dead | 0—Dead |

Abbreviations: ECOG, Eastern Cooperative Oncology Group

**Table S2** Categorization of tumor subtype

| **Tumor subtype for GPA** | **Receptor status at baseline** |
| --- | --- |
| HER2+ | ER-, PR- and HER2+ |
| Basal | ER-, PR- and HER2- |
| Luminal A | ER+ and/or PR+ and HER2- |
| Luminal B | ER+ and/or PR+ and HER2+ |

Abbreviations: ER, estrogen receptor; GPA, Graded Prognostic Assessment; HER2, human epidermal receptor type 2; PR, progestin receptor
